# Stagnation point flow of hybrid nanofluid flow passing over a rotating sphere subjected to thermophoretic diffusion and thermal radiation

**DOI:** 10.1038/s41598-023-46353-z

**Published:** 2023-11-04

**Authors:** Khalid Abdulkhaliq M. Alharbi, Muhammad Bilal, Aatif Ali, Sayed M. Eldin, Amal F. Soliman, Mati Ur Rahman

**Affiliations:** 1 Mechanical Engineering Department, College of Engineering, Umm Al-Qura University, Makkah, 24382 Kingdom of Saudi Arabia; 2 Sheikh Taimur Academic Block-II, Department of Mathematics, University of Peshawar, Peshawar, Khyber Pakhtunkhwa 25120 Pakistan; 3 School of Mathematical Sciences, Jiangsu University, Zhenjiang, 212013 Jiangsu China; 4 Faculty of Engineering, Center of Research, Future University in Egypt, New Cairo, 11835 Egypt; 5 Department of Mathematics, College of Arts and Sciences, Prince Sattam Bin Abdulaziz University, Wadi Addawasir, Saudi Arabia; 6 Department of Basic Science, Benha Faculty of Engineering, Benha University, Banha, Egypt; 7 Department of Computer Science and Mathematics, Lebanese American University, Beirut, Lebanon

**Keywords:** Energy science and technology, Mathematics and computing, Nanoscience and technology

## Abstract

The effects of thermal radiation and thermophoretic particles deposition (TPD) on the hybrid nanofluid (HNF) flow across a circling sphere have momentous roles in research and engineering. Such as electrical devices, projectiles, thermal conveyance, sheet production, renewable energy, and nuclear-powered plants. Therefore, the current study presents the stagnation point flow of HNF flows about an orbiting sphere. The HNF is organized with the accumulation of aluminum alloys (AA70772 and AA7075) nanoparticles in the water. The HNF flow model equations are changed into the non-dimensional form of ODEs through the similarity variables and then numerically solved through the parametric simulation. It has been perceived that the significance of the rotation factor boosts the velocity curve, while the flow motion drops with the increasing numbers of AA7072 and AA7075 nanoparticles. Furthermore, the addition of AA7072 and AA70775 nano particulates in water lessens with the temperature profile. The energy distribution rate in case of hybrid nanoliquid enhances from 3.87 to 13.79%, whereas the mass dissemination rate enhances from 4.35 to 11.24% as the nanoparticles concentration varies from 0.01 to 0.03.

## Introduction

Heat and mass transmission mechanisms are used in many manufacturing operations and energy technologies. Such as heat exchangers, desalination; solar thermal systems, drying process, high-pressure systems; internal ignition engines, gasification processes, gas turbines, innovation, renewable energy systems, and the latest technologies^1–3^. Recently, Gul et al.^4^ discussed the impact of magnetic flux on a 2D couple stress HNF's stagnation point flow around a spinning sphere. It was found that increasing the thermal conductivity of a hybrid nanoliquid from 5.8 to 11.947% by raising the range of nanoparticle from 0.01 to 0.02 and increasing the thermal conductivity of a nanoliquid by the same amount from 2.576 to 5.197%. Sabu et al.^5^ numerically investigated the two-phase Buongiorno model for the nanoliquid flow caused by a spiraling rigid disc. It was discovered that alumina nano particulates with a platelet shape exhibit the drag force. Acharya et al.^6^ examined a stream of magnetized HNF flow over a rotating sphere and resolved that the heat transportation is also larger for HNF than for regular nanofluid. Ahammad et al.^7^ and Ramesh et al.^8^ observed the hybrid nanoliquid flow moved towards a spinning sphere with the thermophoresis deposition of particles and heat radiation. It was pragmatic that the heat dissemination rate increases as the radiation component and the number of nano particulates raise. Gangadhar et al.^9^ discoursed the behavior viscous dissipation as HNFs moved to their stagnation point across a spiraling sphere and detected that the heat Biot number amplified energy propagation and that the pace of growth was faster for hybrid nano-suspension. Bilal et al.^10^ used a computational simulation to show how hall current affected the unstable free convection flow induced by a HNF over a permeable extending surface.

To have better thermochemical, rheological, morphological, and optical properties, HNFs are generated by mixing two different categories of nano particulates into the same base fluid. For several reasons, HNFs are better than simple nanofluids, including their broad absorption spectrum, reduced extinctions, low-pressure drop, reduced frictional loss, and pumping capacity^11^. Solar collectors, electronic components thermal control, solar energy thermal executives, and engine and automobile cooling are just a few of the applications of HNFs^12,13^. Aluminium alloys are extensively employed in the manufacture, scrutiny, and developing of aircraft and spacecraft parts. Researchers looked at different flow models made of aluminium compound and discovered interesting thermal conveyance behavior because AA7072 and AA7075 aluminium alloys have better heat transfer features^14^. Recently, Tlili et al.^15^ have calculated the 3D magnetohydrodynamic (MHD) flow of a HNF encompassing AA7072 + AA7075 NPs of aluminum alloys through an extended plane with slip effects and irregular thickness. Adnan et al.^16^ have examined heat transfer through a tetra-nanoliquid flow across stretching channel. Madhukesh et al.^17^ calculated the flow water-based HNF across an irregular extending sheet and perceived that the flow rate boosts for larger values of the curvature factor. Rekha et al.^18^ utilized a suspended solution of aluminum compounds as tiny particles in water to study the impact of a heat source and sink on nanoliquid flow within a cone, plate, and wedge. It was discovered that the fluid flow over a wedge and plate exhibits better heat conveyance for enhanced heat source/sink coefficients. Waqas et al.^19^ described the role of the melting process and magnetic field for the thermal transportation of blood-based HNF with various nano-sized aluminum alloy particulates in a rotating channel. Tlili et al.^15^ employed the AA7072 + AA7075 NPs in methanol fluid to study a 3-D MHD flow of HNF through an extended plane of irregular width with slip impacts. The findings indicated that the HNF's rate of energy transmission is significantly higher than that of the nanoliquid. Houssem et al.^20^ and Puneeth et al.^21^ described the flow of HNF and gyrotactic microorganisms subject to the characteristics of Casson nanofluid. Sohut et al.^22^ testified the water-based copper-alumina HNF in a tilted cylinder. Murtaza et al.^23^ described the energy transfer within a warmed stretching sheet using an engine oil-based nanoliquid flow made up of cobalt ferrite nanoparticles. It was exposed that the rise of the NPs' volume fraction and permeability factor leads the ambient temperature profile to rise significantly. Some recent results may exist regarding base fluid in Ref.^24–27^.

For an extensive range of applications, such as thermal governance, spectroscopy, energy-conversion, and optoelectronics technology, the effect of thermal radiation is necessary^28^. Nanostructured components i.e. photonic structures of architecture provide new prospects for modifying the infrared thermal spectrum and enhancing the functionality of thermal devices^29^. Recently, several researchers have documented the topic of fluid flow under the significance of heat and thermal radiation^30–33^. Alqahtani et al.^34,35^ examined the enhancement of energy dissemination through ternary HNF flow over a straight surface. The effect of heat radiation was thought to boost the energy graph. Elattar et al.^36^ deliberated the flow of HNF through a permeable thin flexible sheet and concluded that the consequence of the velocity index improves the velocity profile. Alrabaiah et al.^37^ described the time-fractional electro-osmotic flow of a thermal radiation-influenced Brinkman-type digging nanoliquid containing clay nanoparticles.

TPD is the most fundamental techniques for moving small particles over temperature gradient. The TPD phenomenon is important to both electrical engineering and aero-solution. In the suggested model, energy and mass transfer through the HNF flows across a spinning sphere with TPD is examined^38^. Abbas et al.^39^ deliberated the flow of a hybrid nanoliquid over an infinite spinning disc with TPD, variable thermal conductivity. It was determined that raising the Forchheimer and Darcy parameters results in a decrease in fluid velocity. Shah et al.^40^ examined the impact of TPD on the flow characteristics of a second-grade fluid under the upshot of variable viscosity. It was found that the mass profile drops with the effect of thermophoretic factor. Moatimid et al.^41^ investigated at the symmetrical peristaltic motion of microbes in a Rabinowitsch fluid with the TPD in a horizontal tube.

In the practical problems of engineering, researchers often face strong nonlinear BVPs (boundary value problems), which are not easily solved. Other numerical convergence, such as RK4, Newton–Raphson and bvp4c is delicate to the initial guesses. Therefore, the purpose of this method is to encounter the high order nonlinear boundary value problems in diverse fields of nonlinear mechanics, bifurcation problems and thermo-fluids with low computational cost^42^. Chandrasekhar^43^ and Ambarzsumian^44^ was the first to present the PCM and have changed the BVP to Cauchy problem. This strategy was further utilized for the numerically assessment of neutron conveyance hypothesis^45^, wave production, and chemical reactors^46^. Grigoluyk et al.^47^ has employed the PCM approach to the mechanics problems.

Based on the aforementioned literature, it appears that no attempts have been made to study the hybrid nanoliquid made up of aluminum alloys circulating about a rotating sphere at its stagnation point. The effect of thermal radiation and thermophoretic particle deposition on the flow has been also described. The mathematical representation of the flow system is conveyed in the form of nonlinear PDEs that are numerically solved by using the PCM methodology. The aim of the present investigation is to determine the answers to the queries itemized below:

- What is the impact of thermal radiation parameter on the thermal performance of the hybrid nanofluid?
- What is the conduct of AA70772 and AA7075 nanoparticles over fluid temperature, concentration and velocity profiles?
- How the thermophoretic particles deposition factor and chemical reaction affect the concentration profile.

**Figure 1:**
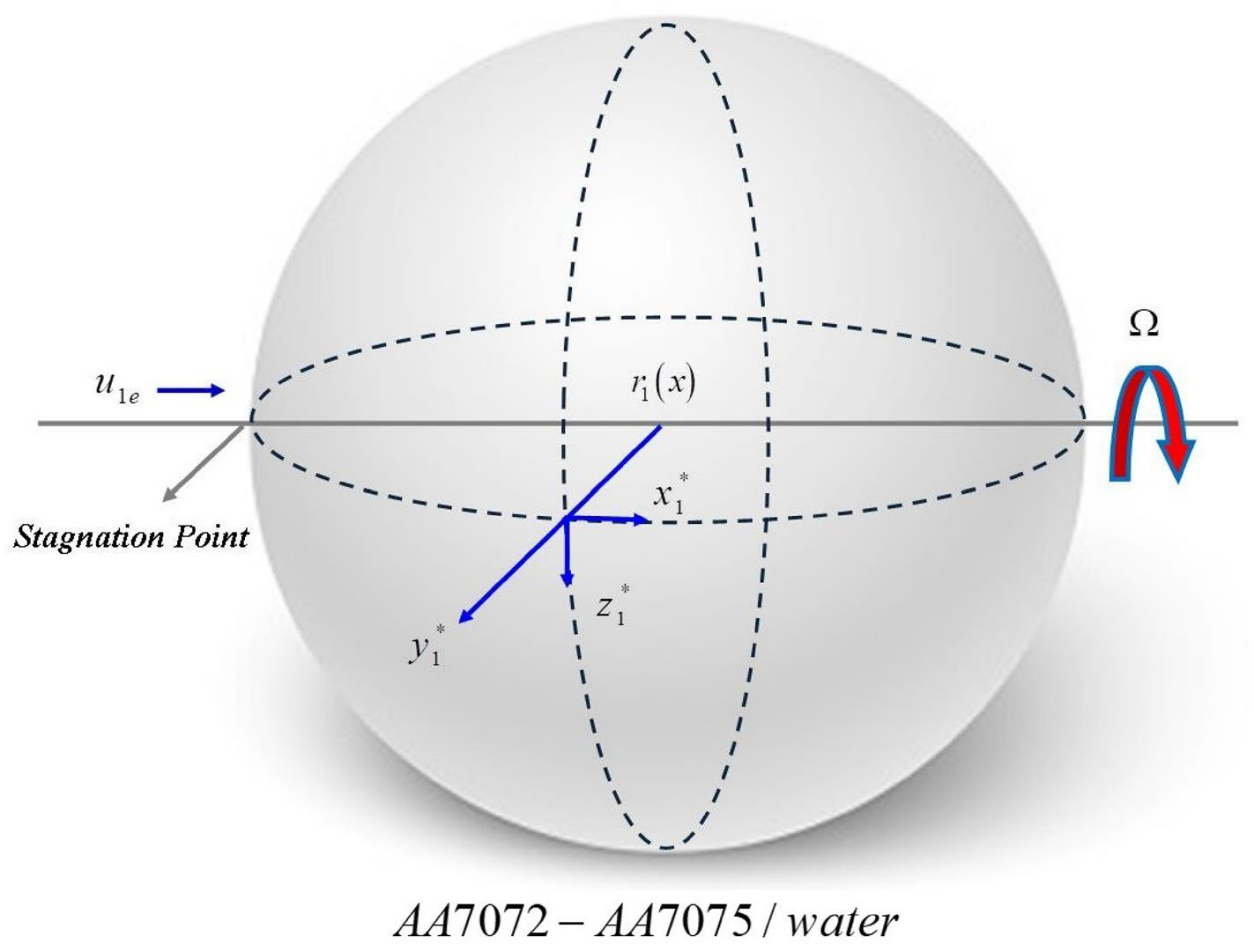
Hybrid nanofluid flow across a spinning sphere.

Furthermore, in “Mathematical formulation”, the hybrid nanoliquid model is formulated and physically discussed along the systematic (Fig. 1). In “Numerical solution”, the solution methodology of the problem is discussed in detail using the PCM approach. “Results and discussion” is the results and discussion section, while the core findings of the present assessment are given in “Conclusions”.

## Mathematical formulation

The stagnation point HNF flow encompassed of AA70772 and AA7075 nanoparticles around an orbiting sphere is deliberated. The *x*-axis and *y*-axis are along the surface and perpendicular to the surface of the sphere as publicized in Fig. 1. An angular velocity of the sphere is mathematically articulated as $\Omega \left( t \right) = \frac{B}{t},\,\,B > 0.$ Moreover, the dissipation term is negligible and the free stream and angular velocity are dependent of time is expressed as $u_{e} \left( {x,\,t} \right) = \frac{xA}{t},\,\,\,\,A > 0$. The concentration and temperature are indicated by $C$ and *T*. The distance from the stagnation point is along *x*-axis. The direction of rotation or span-wise direction is along y-axis. The surface perpendicular to *x* and *y*-axes is dignified as *z*. The governing equations for HNF are stated as^48^:

1 $$\frac{{\partial \left( {ru} \right)}}{\partial x} + \frac{{\partial \left( {rv} \right)}}{\partial y} = 0,$$

2 $$\frac{\partial u}{{\partial t}} + u\left( {\frac{\partial u}{{\partial x}}} \right) + v\left( {\frac{\partial u}{{\partial y}}} \right) = \nu_{hnf} \frac{{\partial^{2} u}}{{\partial y^{2} }} + \frac{1}{r}\left( {\frac{{w^{2} \partial r}}{\partial x}} \right) + u_{e} \left( {\frac{{\partial u_{e} }}{\partial x}} \right) + \frac{{\partial u_{e} }}{\partial t},$$

3 $$\frac{\partial w}{{\partial t}} + u\left( {\frac{\partial w}{{\partial x}}} \right) + v\left( {\frac{\partial w}{{\partial y}}} \right) = \nu_{hnf} \frac{{\partial^{2} w}}{{\partial y^{2} }} - \frac{u}{r}\left( {\frac{w\partial r}{{\partial x}}} \right),$$

4 $$\frac{\partial T}{{\partial t}} + u\left( {\frac{\partial T}{{\partial x}}} \right) + v\left( {\frac{\partial T}{{\partial y}}} \right) = \left( {\frac{{k_{hnf} }}{{\left( {\rho C_{p} } \right)_{hnf} }}} \right)\frac{{\partial^{2} T}}{{\partial y^{2} }} - \left( {\frac{1}{{\left( {\rho C_{p} } \right)_{hnf} }}} \right)\frac{\partial qr}{{\partial y}},$$

5 $$\frac{\partial C}{{\partial t}} + u\left( {\frac{\partial C}{{\partial x}}} \right) + v\left( {\frac{\partial C}{{\partial y}}} \right) = D_{f} \frac{{\partial^{2} T}}{{\partial y^{2} }} - K_{c} \left( {C - C_{0} } \right) - \frac{\partial }{\partial y}\left( {V_{T} \left( {C - C_{\infty } } \right)} \right).$$

Initial conditions:

6 $$u = u_{i} ,\,\,\,\,w = w_{i} ,\,\,\,\,v = v_{i} ,\,\,\,C = C_{i} ,\,\,\,\,T = T_{i} \,\,\,{\text{at}}\,\,\,t = 0.$$

The boundary conditions (BCs) are:

7 $$\left. \begin{gathered} u = 0,\,\,\,w = \Omega \left( t \right)r,\,\,\,v = 0,\,\,\,T = T_{w} ,\,\,\,{\text{at}}\,\,\,\,y = 0,\,\,\, \hfill \\ w \to 0,\,\,T \to T_{\infty } ,\,\,\,u = u_{e} \to \frac{Ax}{t},\,\,\,\,\,C \to C_{\infty } \,\,\,{\text{as}}\,\,\,y \to \infty . \hfill \\ \end{gathered} \right\}$$

The thermophoretic velocity and heat radiation are signified by $V_{T}$ and $qr$ is expressed as^49^.

8 $$V_{T} = - \frac{{K_{2}^{*} \nu_{f} }}{{T_{r} }}\frac{\partial T}{{\partial y}},\,\,\,qr = - \frac{16}{3}\left( {\frac{{\sigma^{*} T_{\infty }^{3} }}{{k^{*} }}} \right)\left( {\frac{\partial T}{{\partial y}}} \right).$$

Here, $\frac{1}{{T_{r} }}$, $K_{2}^{*}$ and $qr$ is the reference temperature, thermophoretic factor, and radiation term.

By incorporating Eq. (8) in Eq. (4), we get:

9 $$\frac{\partial T}{{\partial t}} + v\left( {\frac{\partial T}{{\partial y}}} \right) + u\left( {\frac{\partial T}{{\partial x}}} \right) = \left( {\frac{{k_{hnf} }}{{\left( {\rho C_{p} } \right)_{hnf} }} + \frac{{\left( {\sigma^{*} T_{\infty }^{3} } \right)16}}{{3k^{*} \left( {\rho C_{p} } \right)_{hnf} }}} \right)\frac{{\partial^{2} T}}{{\partial y^{2} }},$$

To transform Eq. (1)–(5) and Eq. (6) into a non-dimensional system of ODE, we use the following relations:

10 $$\left. \begin{gathered} x^{*} \approx r,\,\,\frac{\partial r}{{\partial x}} = 1,\,\,\,w = \frac{Bx}{t}g\left( \eta \right),\,\,\eta = \left( {\nu_{f} t} \right)^{ - 1} y,\,\,\,\psi = Ax\left( {\nu_{f} t^{ - 1} } \right)^{\frac{1}{2}} f\left( \eta \right), \hfill \\ \varphi \left( \eta \right) = \frac{{C - C_{\infty } }}{{C_{w} - C_{\infty } }},\,\,\,\,\,\theta \left( \eta \right) = \frac{{T - T_{\infty } }}{{T_{w} - T_{\infty } }}. \hfill \\ \end{gathered} \right\}$$

Table 1 and 2 exhibit the experimental values utilized in the approximation of the mathematical model and problem.

**Table 1:** The experimental values of water and nano particulates^18^.

	$\rho (kg/m^{3} )$	$C_{p} (j/kgK)$	$k(W/mK)$
Water	997.1	_4179_	0.613
AA7075	_2810_	_960_	173
AA7072	_2720_	_893_	222

**Table 2:** Mathematical model for the hybrid nanofluid $\left( {\phi_{1} = \phi_{AA7072} ,\,\,\,\phi_{2} = \phi_{AA7075} } \right)$^18^.

Properties	
Viscosity	$\frac{{\mu_{hnf} }}{{\mu_{f} }} = \left( {1 - \phi_{AA7072} } \right)^{ - 2.5} \left( {1 - \phi_{AA7075} } \right)^{ - 2.5}$
Density	$\rho_{hnf} = \left( {\phi_{1} \rho_{AA7072} + \left( {1 - \phi_{1} } \right)\rho_{f} } \right) + \phi_{2} \rho_{{_{AA7075} }} ,$
Thermal capacity	$(\rho C_{p} )_{hnf} = \phi_{2} (\rho C_{p} )_{AA7075} + \left( {\left( {1 - \phi_{1} } \right)(\rho C_{p} )_{f} + \phi_{1} (\rho C_{p} )_{AA7072} } \right)\left( {1 - \phi_{2} } \right)$
Thermal conductivity	$\frac{{k_{hnf} }}{{k_{nf} }} = \frac{{2\phi_{2} \left( {\frac{{k_{AA7075} }}{{k_{AA7075} - k_{nf} }}} \right)\ln \left( {\frac{{k_{AA7075} + k_{nf} }}{{2k_{nf} }}} \right) + \left( {1 - \phi_{2} } \right)}}{{2\phi_{2} \left( {\frac{{k_{nf} }}{{k_{AA7075} - k_{nf} }}} \right)\ln \left( {\frac{{k_{AA7075} + k_{nf} }}{{2k_{nf} }}} \right) + \left( {1 - \phi_{2} } \right)}}$ $\frac{{k_{nf} }}{{k_{f} }} = \frac{{2\phi_{1} \left( {\frac{{k_{AA7072} }}{{k_{AA7075} - k_{f} }}} \right)\ln \left( {\frac{{k_{AA7072} + k_{f} }}{{2k_{nf} }}} \right) + \left( {1 - \phi_{1} } \right)}}{{2\phi_{1} \left( {\frac{{k_{f} }}{{k_{AA7072} - k_{f} }}} \right)\ln \left( {\frac{{k_{AA7072} + k_{f} }}{{2k_{f} }}} \right) + \left( {1 - \phi_{1} } \right)}}$

By employing Eq. (10) in Eqs. (1)–(5), we get:

11 $$f{\prime \prime \prime } + A_{1} A_{2} \left\{ {f{\prime } - Af^{{{\prime }2}} + f{\prime \prime }\left( {\frac{\eta }{2}} \right) + A\left( {\lambda g^{2} + 1} \right) - 1} \right\} = 0,$$

12 $$g{\prime \prime } + A_{1} A_{2} \left\{ {g(1 - 2Af^{\prime}) + g^{\prime}\left( {\frac{\eta }{2} + fA} \right)} \right\} = 0,$$

13 $$\frac{1}{{A_{3} }}\left( {\frac{{k_{knf} }}{{k_{nf} }} + \frac{4}{3}Rd} \right)\theta {\prime \prime } + Pr\theta^{\prime}\left( {\frac{\eta }{2} + fA} \right) = 0,$$

14 $$\begin{gathered} \varphi^{\prime\prime} + Sc\varphi^{\prime}\left( {\frac{\eta }{2} + fA} \right) - \tau Sc\left( {\varphi \theta^{\prime \prime} + \varphi^{\prime}\theta^{\prime}} \right) - Sc\,Kr\varphi \,\left( \eta \right) = 0. \hfill \\ \hfill \\ \end{gathered}$$

The reduced BCs are:

15 $$\begin{gathered} f{\prime }\left( \eta \right) = 1,\,\,\,\,\,g\left( \eta \right) = 1,\,\,\,\,\,f\left( \eta \right) = 0,\,\,\,\theta \left( \eta \right) = 1,\,\,\,\,\varphi \left( \eta \right) = 1,\,\,\,{\text{at}}\,\,\,\,\eta = 0, \hfill \\ \,f{\prime }\left( \eta \right) = 0,\,\,\,g\left( \eta \right) = 0,\,\,\,\,\varphi \left( \eta \right) = 0,\,\,\,\,\theta \left( \eta \right) = 0\,\,\,{\text{as}}\,\,\,\,\eta \to \infty . \hfill \\ \end{gathered}$$

where,

$$\begin{gathered} A_{1} = \left( {1 - \phi_{AA7072} } \right)^{2.5} \left( {1 - \phi_{AA7075} } \right)^{2.5} ,\,\,\,A_{2} = \left( {1 - \phi_{AA7075} } \right)\left( {\phi_{AA7072} \frac{{\rho_{{_{AA7072} }} }}{{\rho_{f} }} + \left( {1 - \phi_{AA7072} } \right)} \right) + \phi_{2} \frac{{\rho_{AA7075} }}{{\rho_{f} }},\,\, \hfill \\ A_{3} = \left( {1 - \phi_{AA7075} } \right)\left\{ {\left( {1 - \phi_{AA7072} } \right) + \phi_{AA7072} \frac{{(\rho C_{p} )_{AA7075} }}{{(\rho C_{p} )_{f} }}} \right\} + \phi_{AA7075} \frac{{(\rho C_{p} )_{AA7075} }}{{(\rho C_{p} )_{f} }}, \hfill \\ \end{gathered}$$

The physical parameters derived from the above equations re given in the following list:

**Table:** 

List of dimensionless parameters		
Parameters	Mathematical expressions	Symbols
Schmidt number	$Sc = \frac{{\nu_{f} }}{{D_{f} }}$	*Sc*
Rotation parameter	$\lambda = \left( \frac{B}{A} \right)^{2}$	$\lambda$
Thermophoretic factor	$\tau = \frac{{ - K_{2}^{*} \left( {T_{w} - T_{\infty } } \right)}}{{T_{r} }}$	$\tau$
Prandtl number	$Pr = \left( {\frac{{\mu_{f} C_{p} }}{{k_{f} }}} \right)$	*Pr*
Thermal radiation	$Rd = \frac{{4\sigma^{*} T_{\infty }^{3} }}{{kk^{*} }}$	*Rd*

The physical quantities are^48,49^:

16 $$Cf_{z} = \frac{{\mu_{hnf} }}{{\rho_{f} u_{e}^{2} }}\left( {\frac{\partial u}{{\partial y}}} \right)_{y = 0} \to \left( {\text{Re}} \right)^{\frac{1}{2}} Cf_{z} = \frac{{ - g\left( 0 \right)\lambda^{\frac{1}{2}} }}{{\sqrt a \,A_{1} }},\,\,Cf_{x} = \frac{{\mu_{hnf} }}{{\rho_{f} u_{e}^{2} }}\left( {\frac{\partial u}{{\partial y}}} \right)_{y = 0} \to \left( {\text{Re}} \right)^{\frac{1}{2}} Cf_{x} = \frac{{f{\prime \prime }\left( 0 \right)}}{{\sqrt a \,A_{1} }}.$$

17 $$\left. \begin{gathered} Nu = \frac{{ - x\left( {\frac{16}{3}\frac{{\sigma^{*} T_{\infty }^{3} }}{{k^{*} }} + k_{hnf} } \right)\left( {\frac{\partial T}{{\partial y}}} \right)_{y = 0} }}{{\left( {T_{w} - T_{\infty } } \right)k_{f} }}\,\,\,\, \to \,\,\,\,\left( {Re} \right)^{{\frac{ - 1}{2}}} Nu = \left( {\frac{{\theta {\prime }\left( 0 \right)}}{\sqrt a }} \right)\left( {\frac{{k_{hnf} }}{{k_{f} }} + \frac{4}{3}Rd} \right), \hfill \\ Sh = \frac{{ - xD_{f} }}{{\left( {C_{w} - C_{\infty } } \right)D_{f} }}\left( {\frac{C}{\partial y}} \right)_{y = 0} \to \left( {Re} \right)^{{\frac{ - 1}{2}}} Sh = \sqrt a \,\,\varphi {\prime }\left( 0 \right). \hfill \\ \end{gathered} \right\}$$

## Numerical solution

PCM approach contains of the following steps^50^:

Step 1:

18 $$\left. \begin{gathered} f(\eta ) = \,\aleph_{1} (\eta ),\,\,\,\,f^{\prime}(\eta ) = \,\,\aleph_{2} (\eta ),\,\,\,f^{\prime \prime}(\eta ) = \,\aleph_{3} (\eta ),\,\,g(\eta ) = \aleph_{4} (\eta ),\,\,g^{\prime}(\eta ) = \aleph_{5} (\eta ),\,\,\,\, \hfill \\ \theta (\eta ) = \aleph_{6} (\eta ),\,\,\,\theta^{\prime}(\eta ) = \,\aleph_{7} (\eta ),\,\,\,\,\,\,\varphi (\eta ) = \,\aleph_{8} (\eta ),\,\,\,\,\,\varphi^{\prime}(\eta ) = \,\aleph_{9} (\eta ). \hfill \\ \,\,\, \hfill \\ \end{gathered} \right\}$$

By putting Eq. (22) in Eqs. (15)-(19), we get:

19 $$\aleph_{3}^{\prime } (\eta ) + A_{1} A_{2} \left\{ {\aleph_{2} (\eta ) - A\left( {\aleph_{2} (\eta )} \right)^{2} + \left( {\frac{\eta }{2}} \right)\aleph_{3} (\eta ) + A\left( {\lambda \left( {\aleph_{4} (\eta )} \right)^{2} + 1} \right) - 1} \right\} = 0,$$

20 $$\,\aleph_{5}^{\prime } (\eta ) + A_{1} A_{2} \left\{ {\aleph_{4} (\eta )(1 - 2A\aleph_{2} (\eta )) + \aleph_{5} (\eta )\left( {\frac{\eta }{2} + A\aleph_{1} (\eta )} \right)} \right\} = 0,$$

21 $$\frac{1}{{A_{3} }}\left( {\frac{{k_{knf} }}{{k_{nf} }} + \frac{4}{3}Rd} \right)\aleph_{7}^{\prime } (\eta ) + Pr\aleph_{7} (\eta )\left( {\frac{\eta }{2} + \aleph_{1} (\eta )A} \right) = 0,$$

22 $$\,\aleph_{9}^{\prime } (\eta ) + Sc\,\aleph_{9} (\eta )\left( {\frac{\eta }{2} + \,\aleph_{1} (\eta )A - \,\,\tau \aleph_{7} (\eta )} \right) - \tau Sc\left( {\,\aleph_{8} (\eta )\,\aleph_{7}^{\prime } (\eta )} \right) - Sc\,Kr\aleph_{8} (\eta )\, = 0,$$

with the corresponding boundary conditions.

23 $$\left. \begin{gathered} \aleph_{1} (\eta ) = 1,\,\,\,\,\aleph_{2} (\eta ) = 0,\,\,\,\,\aleph_{4} (\eta ) = \aleph_{6} (\eta ) = \aleph_{8} (\eta ) = 1,\,\,\,{\text{at}}\,\,\,\,\eta = 0, \hfill \\ \aleph_{2} (\eta ) = 0,\,\,\,\,\aleph_{4} (\eta ) = \aleph_{6} (\eta ) = \aleph_{8} (\eta ) = 0,\,\,\,{\text{as}}\,\,\,\,\eta \to \infty . \hfill \\ \end{gathered} \right\}$$

Step 2: Introducing parameter *p* in Eqs. (19)–(22):

24 $$\aleph_{3}^{\prime } (\eta ) + A_{1} A_{2} \left\{ {\aleph_{2} (\eta ) - A\left( {\aleph_{2} (\eta )} \right)^{2} + \left( {\frac{\eta }{2}} \right)\left( {\left( {\aleph_{3} (\eta ) - 1} \right)p + 1} \right) + A\left( {\lambda \left( {\aleph_{4} (\eta )} \right)^{2} + 1} \right) - 1} \right\} = 0,$$

25 $$\,\aleph_{5}^{\prime } (\eta ) + A_{1} A_{2} \left\{ {\aleph_{4} (\eta )(1 - 2A\aleph_{2} (\eta )) + \left( {\frac{\eta }{2} + A\aleph_{1} (\eta )} \right)\left( {\left( {\aleph_{5} (\eta ) - 1} \right)p + 1} \right)} \right\} = 0,$$

26 $$\frac{1}{{A_{3} }}\left( {\frac{{k_{knf} }}{{k_{nf} }} + \frac{4}{3}Rd} \right)\aleph_{7}^{\prime } (\eta ) + Pr\left( {\frac{\eta }{2} + \aleph_{1} (\eta )A} \right)\left( {\left( {\aleph_{7} (\eta ) - 1} \right)p + 1} \right) = 0,$$

27 $$\,\aleph_{9}^{\prime } (\eta ) + Sc\,\left( {\frac{\eta }{2} + \,\aleph_{1} (\eta )A - \,\,\tau \aleph_{7} (\eta )} \right)\left( {\left( {\aleph_{9} (\eta ) - 1} \right)p + 1} \right) - \tau Sc\left( {\,\aleph_{8} (\eta )\,\aleph_{7}^{\prime } (\eta )} \right) - Sc\,Kr\aleph_{8} (\eta )\, = 0.$$

Step 3: Differentiating ‘*p*’

By differentiating Eqs. (24)–(27) w. r. t parameter *p*, we get:

28 $$V^{\prime} = AV + R,$$

and

29 $$\frac{{d\zeta_{i} }}{d\tau }$$

where *i* = 1*,* 2*, ………*11*.*

## Step 4: Apply the superposition rule

30 $$V = aU + W,$$

For every element, solve the Cauchy problems as:

31 $$\left. \begin{gathered} U = aU, \hfill \\ W^{\prime} = AW + R, \hfill \\ \end{gathered} \right\}$$

By placing Eq. (32) in Eq. (31):

32 $$(aU + W)^{\prime} = (aU + W)A + R,$$

Step 5: Resolving the Cauchy problems

By employing numerical implicit scheme as:

33 $$\frac{{U^{i + 1} - U^{i} }}{\Delta \eta } = AU^{i + 1} \,\,\,\& \,\,\,\,\frac{{W^{i + 1} - W^{i} }}{\Delta \eta } = AW^{i + 1} .$$

Finally, we acquire the iterative form as:

34 $$\left. \begin{gathered} U^{i + 1} = (I - \Delta \eta A)^{ - 1} U^{i} , \hfill \\ W^{i + 1} = (I - \Delta \eta A)^{ - 1} (W^{i} + \Delta \eta R). \hfill \\ \end{gathered} \right\}$$

## Results and discussion

The HNF comprised of AA70772 & AA7075 stagnation point flow about an orbiting sphere is calculated in the current study. By using the default values of the non-dimensional parameters $Rd = 0.1,$ $A = 0.3,$ $Sc = 0.2,$ $Kc = 0.5,$ and $\phi_{1} = \phi_{2} = 0.01.$

**Figure 2:**
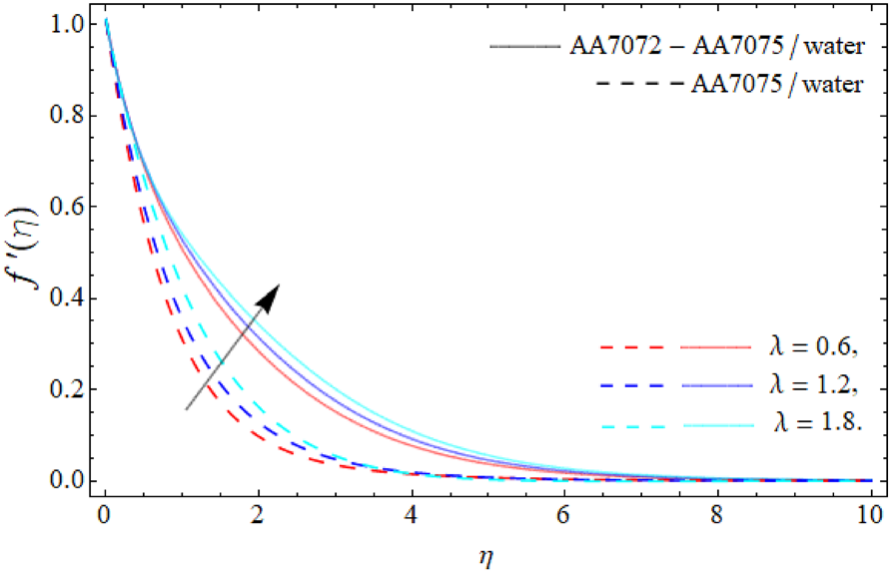
Velocity $f^{\prime}\left( \eta \right)$ curve versus the rotational parameter $\left( \lambda \right)$.

**Figure 3:**
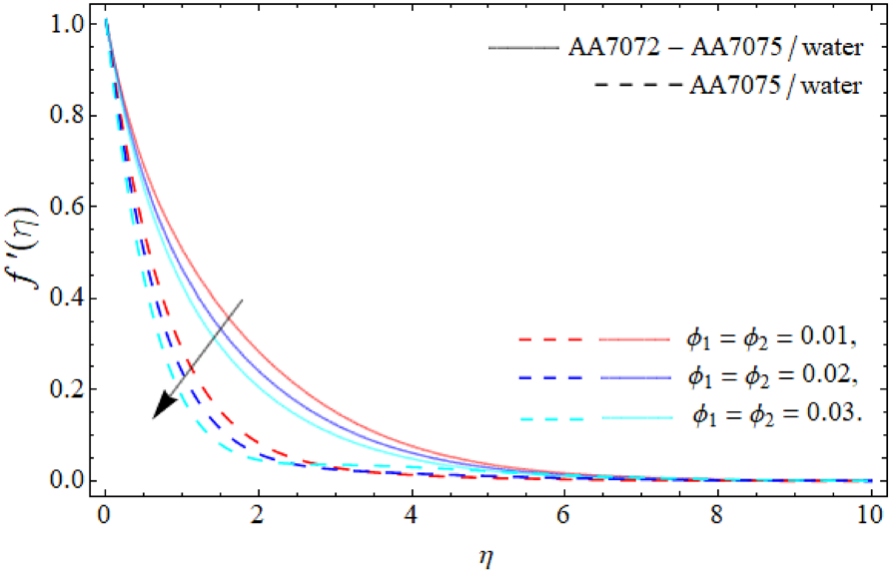
Velocity $f^{\prime}\left( \eta \right)$ curve versus the nanoparticles $\left( {\phi_{1} = \phi_{2} } \right)$.

**Figure 4:**
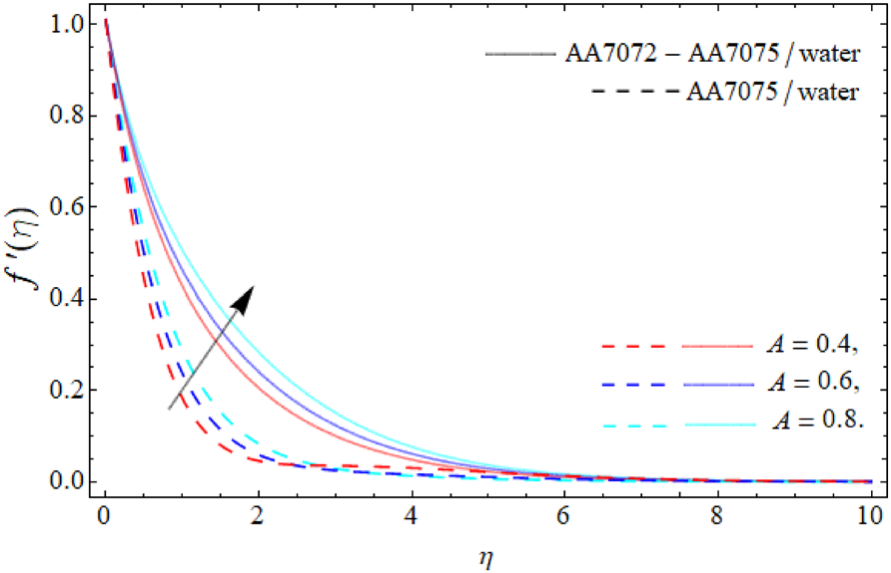
Velocity $f^{\prime}\left( \eta \right)$ curve versus the acceleration factor *A*.

**Figure 5:**
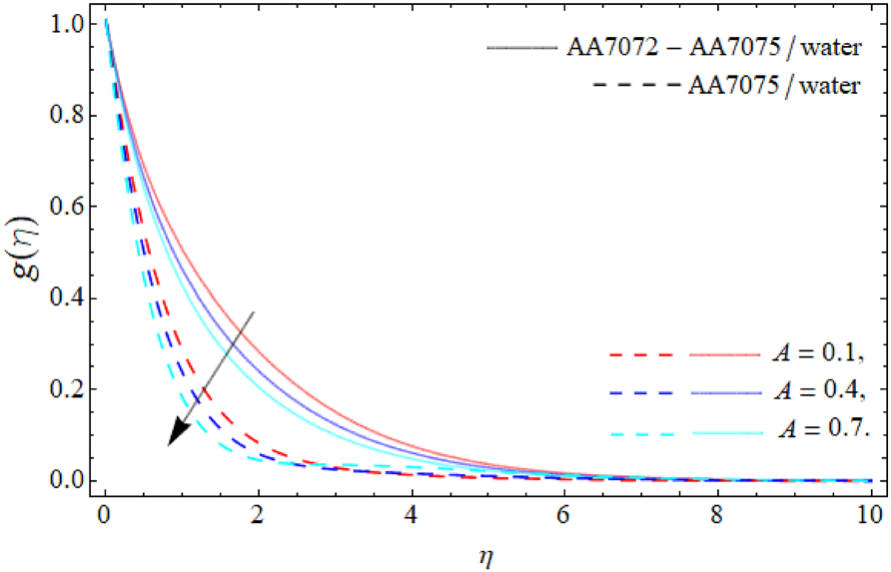
Velocity $g\left( \eta \right)$ curve versus the acceleration term *A*.

Figures 2, 3, 4 and 5 display the consequences of dimensionless factors (rotational term $\lambda ,$ AA70772 & AA7075 nano particulates $\left( {\phi_{1} = \phi_{2} } \right)$ and acceleration factor *A*) on the velocity $\left( {f^{\prime}\left( \eta \right),\,\,g\left( \eta \right)} \right).$ It has been detected that the significance of rotation term boosts the velocity profile, because the spinning of sphere induced the fluid motion, as a result, the flow motion enhances (Fig. 2). Figure 3 exposes that the flow motion declines with the rising mounting values of AA7075 & AA7072. Comparatively, the heat absorbing capability and density of water is lighter than the aluminum alloys; therefore the rising numbers of AA7075 & AA7072 reduce the fluid flow as well as the boundary layer thickness as seen in Fig. 3. It can be noticed from Figs. 4 and 5 that the axial velocity $\left( {f^{\prime}\left( \eta \right)} \right)$ of fluid enriches, while the radial velocity $\left( {g\left( \eta \right)} \right)$ of fluid diminishes with the influence of acceleration factor *A.*

**Figure 6:**
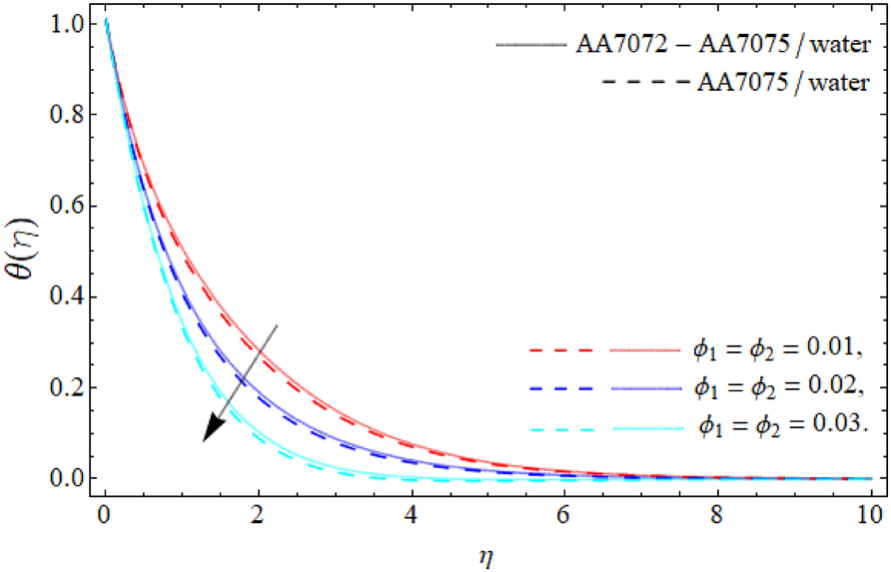
Energy curve $\theta \left( \eta \right)$ versus the nanoparticles $\left( {\phi_{1} = \phi_{2} } \right)$.

**Figure 7:**
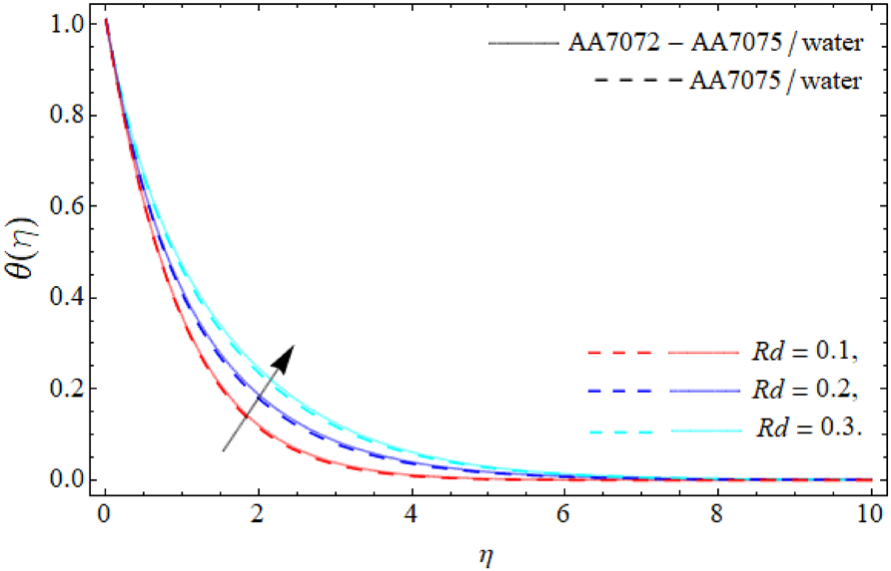
Energy curve $\theta \left( \eta \right)$ versus $\left( {Rd} \right)$.

**Figure 8:**
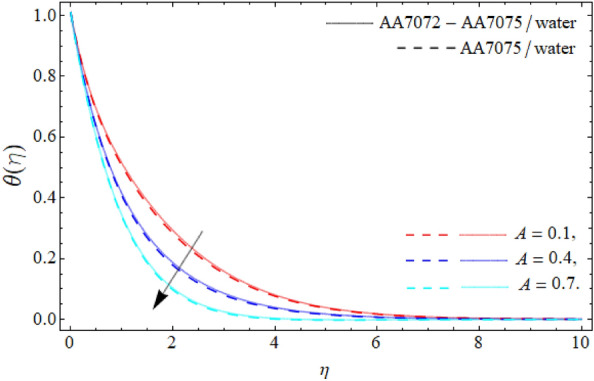
Energy curve $\theta \left( \eta \right)$ versus the acceleration factor $\left( A \right)$.

Figures 6, 7 and 8 disclose the significances of AA70772 & AA7075 nano particulates $\left( {\phi_{1} = \phi_{2} } \right)$, thermal radiation $\left( {Rd} \right)$ and acceleration factor $\left( A \right)$ on the energy field $\theta \left( \eta \right).$ Figure 6 expresses that temperature curve lessens with the consequence of AA7072 and AA70775 nano particulates $\left( {\phi_{1} = \phi_{2} } \right).$ Physically, the heat absorbing capability and density of water is lighter than the aluminum alloys, and the boundary layer thickness rises with the rising numbers of nanoparticles; therefore the mounting quantity of AA7072 and AA7075 in water make the fluid molecules more denser and also amplify the heat absorbing capability of base fluid, that’s why, the energy outlines falls due the effect of aluminum alloys as seen in Fig. 6. On the other side, energy field of hybrid nanoliquid accelerates with rising influence of thermal radiation as publicized in Fig. 7. Physically, *Rd* is the discharge waves (of electromagnetic) from matter that that has higher temperature than absolute zero, that’s why, the upshot of *Rd* boosts the energy profile. Figure 8 reveals the impacts of the acceleration factor $\left( A \right)$ on the energy field $\theta \left( \eta \right).$ It has been perceived that the temperature field of the HNF drops with the action of the acceleration factor.

**Figure 9:**
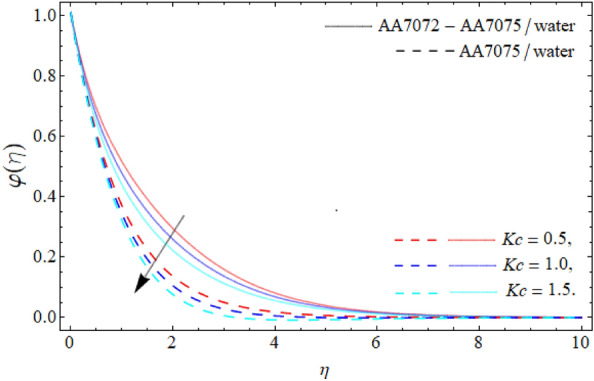
Mass profile $\varphi \left( \eta \right)$ versus *Kc*.

**Figure 10:**
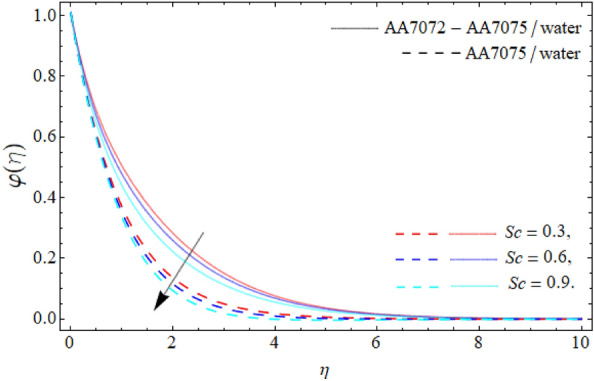
Mass outline $\varphi \left( \eta \right)$ versus the *Sc*.

**Figure 11:**
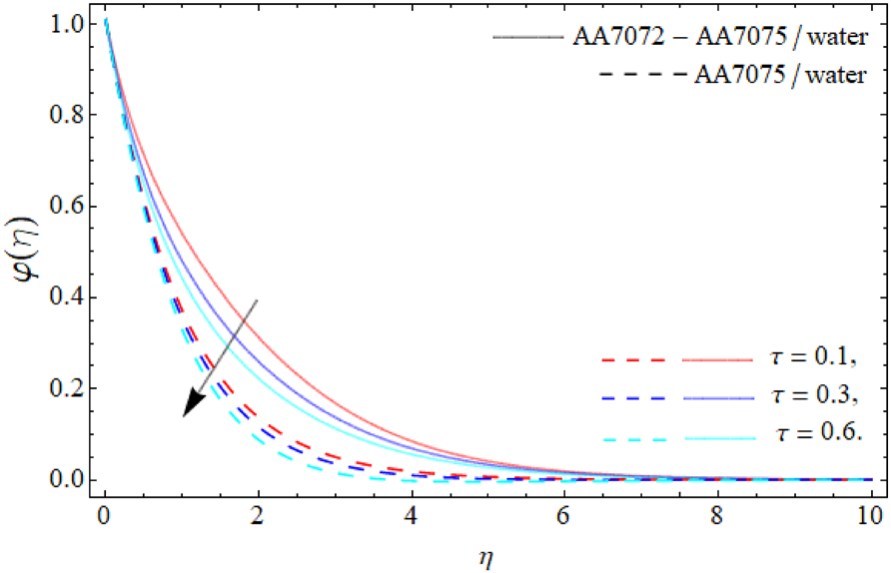
Mass outline $\varphi \left( \eta \right)$ versus the thermophoretic particles deposition parameter $\tau$.

Figures 9 and 10 demonstrate the behavior of *Kc* and *Sc* on the mass profile $\varphi \left( \eta \right)$. From Fig. 9, it can be perceived that the mass communication rate falls with the variation of *Kc*. Physically, the chemical transfiguration happens as a consequence of a chemical reaction. Therefore, the impact of *Kc* falls the mass profile as specified in Fig. 9. The diffusion ratio of mass is contrariwise correlated to the Schmidt number, whereas the viscosity is directly proportionate to the *Sc*, so the impact of *Sc* reduces the mass curve; therefore, the variation of Schmidt number reduce the mass transference rate as exhibited in Fig. 10. Figure 11 reveals the influence of $\tau$ on the mass profile $\varphi \left( \eta \right)$. It can be perceived that the fluid concentration profile drops with the variation of thermophoretic particles deposition parameter $\tau$. Physically, the imposing of particle deposition has a declining influence on the rate of mass transmissions respectively.

Table 3 determines the comparative valuation of the current results with the existing works by varying *A* (acceleration factor)*.* The outcomes of the examination specify a precise comparison. Table 4 elucidates the Statistical results of skin friction, Nusselt number, and Sherwood number. It can be perceived that the upshot of the acceleration and rotation factors increases skin friction. Furthermore, the impact of radiation improves the energy transfer rate. Table 5 illustrates the percentage $\left( \% \right)$ assessment for energy and mass transfer rate with the variation of $\lambda$, *Sc* and $\phi$. The energy distribution rate in case of hybrid nanoliquid enhances from 3.87% to 13.79%, whereas the mass dissemination rate enhances form 4.35% to 11.24% as the nanoparticles concentration varies from 0.01 to 0.03.

**Table 3:** Comparative valuation of the current results with the existing literature.

	Malvandi^48^	Ramesh et al.^8^	Present work	Malvandi^48^	Ramesh et al.^8^	Present work
A	$f^{\prime\prime}\left( 0 \right)$	$f^{\prime\prime}\left( 0 \right)$	$f^{\prime\prime}\left( 0 \right)$	$- \theta^{\prime}\left( 0 \right)$	$- \theta^{\prime}\left( 0 \right)$	$- \theta^{\prime}\left( 0 \right)$
0.5	0.79923	0.79931	0.7995612	0.467658	0.467666	0.4676826
1.0	1.2838	1.28242	1.2823920	0.589537	0.589546	0.58956452
2.0	1.9182	1.91855	1.9187631	0.779536	0.779548	0.77959170

**Table 4:** Statistical results of engineering importance quantities.

$\lambda$	*Rd*	*Sc*	$\tau$	*A*	$C_{fx}$	$C_{fz}$	*Nu*	*Sh*
0.3	0.4	0.5	0.1	1.0	1.1165312	0.6615861	5.6129832	0.6280846
0.6					1.3584310	1.4230629	5.6385738	0.6349927
0.9					1.5945852	2.0269917	5.6629748	0.64149278
	0.4				1.11653815	0.6615862	5.4294832	0.71503721
	0.8				–	–	5.6129891	0.62809423
	1.2				–	–	5.7132041	0.57510921
		0.5			–	–	5.6129627	0.8280092
		1.0			–	–		1.09211072
		1.5			–	–		1.55549370
			0.1		–	–		0.72809473
			0.2		–	–		1.27781722
			0.3		–	–		2.10892832
				1.0	0.0968971	0.7686029	5.3237738	0.61510923
				2.0	1.1165361	0.6615462	5.6129827	0.72809822
				3.0	2.0806748	1.3920863	5.6898873	0.72820981

**Table 5:** Percentage $\left( \% \right)$ assessment for energy and mass transfer rate with the variation of $\lambda$, *Sc* and $\phi$.

Parameters	Values	$\left( {\frac{{Nu_{{\phi_{\,0.03} }} - Nu_{{\phi_{\,0.01} }} }}{{Nu_{{\phi_{\,0.01} }} }}} \right) \times 100$ (%)	$\left( {\frac{{Sh_{{\phi_{\,0.03} }} - Sh_{{\phi_{\,0.01} }} }}{{Sh_{{\phi_{\,0.01} }} }}} \right) \times 100$ (%)
$\lambda$	0.1	0.270274	0.880132
	0.3	0.310348	0.880188
	0.5	0.351928	0.921988
*Sc*	0.5	0.260864	0.960956
	1.0	0.292669	0.931014
	1.5	0.313279	0.948986
$\phi_{1} ,\,\,\,\phi_{2}$	0.00	0.7346125	0.997972
	0.01	3.873281	4.357289
	0.02	7.993707	8.394718
	0.03	13.79315	11.24632

## Conclusions

We have assessed the consequences of chemical reaction, thermal radiation and TPD on the HNF flow over a spinning sphere. The study presents the stagnation point flow of HNF flows about an orbiting sphere. The HNF is systematized with the accumulation of AA70772 & AA7075 nanoparticles in the water. The HNF flow model equations are changed into the non-dimensional form of ODEs through the similarity variables and then numerically cracked through the PCM. The key deductions are:

- The significance of the rotation term boosts the velocity profile, while the flow motion decays with the mounting values of AA7072 & AA7075.
- The energy distribution rate in case of hybrid nanoliquid enhances from 3.87% to 13.79%, whereas the mass dissemination rate enhances form 4.35% to 11.24% as the nanoparticles concentration varies from 0.01 to 0.03.
- The axial velocity $\left( {f^{\prime}\left( \eta \right)} \right)$ of fluid enriches, while the radial velocity $\left( {g\left( \eta \right)} \right)$ of fluid diminishes with the impact of acceleration factor *A.*
- The temperature curve lessens with the consequence of AA7072 & AA70775 nano particulates $\left( {\phi_{1} = \phi_{2} } \right).$
- The energy field of hybrid nanoliquid accelerates with the rising influence of thermal radiation, while the impacts of the acceleration factor $\left( A \right)$ drop the energy field $\theta \left( \eta \right).$
- The effect of *Kc*, *Sc* and thermophoretic particles deposition parameter $\tau$ declines the concentration profile $\varphi \left( \eta \right)$.
- The current model can be modified by considering other types of fluid rather than water and also using different sorts of nanoparticles. It can also be solved through other numerical and analytical procedures.

## Data Availability

All data used in this manuscript is present within the article.
